# Fractional exhaled nitric oxide and eosinophil count in induced sputum to guide the management of children with asthma: a cost-utility analysis

**DOI:** 10.1186/s12890-022-02027-6

**Published:** 2022-06-28

**Authors:** Jefferson Antonio Buendía, Diana Guerrero Patiño, Jorge Mario Sánchez Caraballo

**Affiliations:** 1 Department of Pharmacology and Toxicology, School of Medicine, Research Group in Pharmacology and Toxicology (INFARTO), Facultad de Medicina, Universidad de Antioquia, Carrera 51D #62-29, Medellín, Colombia; 2 Hospital Concejo Infantil de Medellin, Medellín, Colombia; 3 Group of Clinical and Experimental Allergy, IPS Universitaria Clinic, University of Antioquia, Medellín, Colombia

**Keywords:** Health economics, Public health, Healthcare

## Abstract

**Introduction:**

Previous evidence has shown that fractional exhaled nitric oxide (FeNO) and eosinophil count in induced sputum (EO) are cost-effective relative to standard of care in guiding the management of children with persistent asthma. There is some doubt as if there are differences between these two biomarkers in terms of costs and benefits. Clarifying this doubt would allow prioritization of the design of clinical practice guidelines. The study aimed to compare in terms of costs and benefits these biomarkers in patients with asthma between 4 and 18 years of age.

**Methods:**

A Markov model was used to estimate the cost-utility of asthma management using FeNO and EO in patients between 4 and 18 years of age. Transition probabilities, cost and utilities were estimated from previously published local studies, while relative risks were obtained from the systematic review of published randomized clinical trials. The analysis was carried out from a societal perspective.

**Results:**

The expected annual cost per patient with EO was US $1376 (CI 95% US $1376–US $1377) and for FeNO was US $1934 (CI 95% US $1333–US $1334), with a difference of US $42.3 between these strategies. The Quality-adjusted life years (QALYs) per person estimated with EO was 0.95 (CI 95% 0.951–0.952) and for FeNO was 0.94 (CI 95% 0.930–0.940), with a difference of 0.01 between these strategies. The NMB with EO was US $4902 (CI 95% 4900–4904) and for FeNO was US $4841 (CI 95% 4839–4843). The incremental cost-effectiveness ratio of EO was $3566 per QALY gained regarding FeNO.

**Conclusion:**

Our study demonstrates that induced sputum-guided management is a strategy cost-effective over FeNO and standard asthma management in Colombia. This evidence should encourage the adoption of any of these techniques to objectively guide the management of children with asthma in routine clinical practice in low-resource settings.

## Introduction

International clinical guidelines recommend the periodic assessment of airway inflammation in patients with asthma as one of the principal strategies to prevent hospitalizations [1]. One strategy for anticipating exacerbations and optimizing the use of biological and corticosteroid drugs is the measure of airway inflammation [2, 3]. Technologies for non-invasive measurement of airway inflammation include fractional exhaled nitric oxide (FeNO) [2, 3]. Nevertheless, a meta-analysis of randomized clinical trials demonstrates that FeNO-guided treatment reduced asthma exacerbations, this test has not been uniformly adopted, especially by developing countries [4, 5]. Another biomarker studied has been the eosinophil count in induced sputum. As with FeNO, tailoring asthma interventions based on sputum eosinophils reduces the frequency of asthma exacerbations (OR 0.36, 95% CI 0.21–0.62), but it is not routinely used in clinical practice. [6]. Sampling success with an adequate sputum sample is achieved in more than 80% of preschooler without any adverse events [7, 8]. One of the main challenges in the adoption of these technologies is the scarce comparative evidence in terms of benefits and costs leading to insufficient links between health technology assessment and decision making by stakeholders [9].

We demonstrated in a previous paper how induced sputum-guided management (EO) is cost-effective for the Colombian health system [10]. This economic model showed that EO was associated with lower cost than standard management based on clinical symptoms with or without spirometry/peak flow (SC) (US $1375 vs. US $1454 average annual cost per patient), and higher quality-adjusted life year or QALY (0.95 vs. 0.92 average per patient). Also, in a previous paper, we show that FeNO was associated with a lower total cost than standard therapy (US $1333 vs. US $1452 average cost per patient) and higher QALYs (0.93 vs. 0.92 average per patient) [5, 11]. FeNO with fast-response chemiluminescence analyzers and flow control devices may make it feasible to measure FeNO with a single breath technique in children as young as 3 years [12]. In this scenario, the clinicians in our country are faced with choosing between FeNO and EOOne way to answer this dilemma is to use a mathematical model to simulate three cohorts of patients, each receiving FeNO-guided management, EO-guided management, and standard management. In the end, evaluate the costs and benefits measured in quality-adjusted life-years gained by each of the alternatives. Clarifying this doubt would allow prioritization in the design of clinical practice guidelines one of the two methods for clinical decision making. The objective of this study is to compare in terms of costs and benefits these two biomarkers in patients with asthma between 4 and 18 years of age.

## Material and methods

### Economic model

**Fig. 1:**
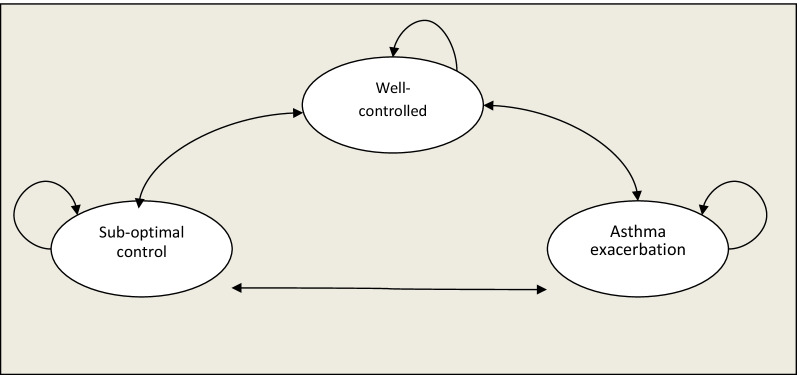
Markov model

To estimate cost and QALY for FeNO, EO, and standard asthma care (low dose inhaled corticosteroids) without the use of FeNO or EO, we use a Markov simulation model. The interventions evaluated in this simulation were adjustment of asthma therapy (stepping up or down inhaled corticosteroid treatment) based on FeNO or sputum eosinophils to adjusting therapy in children between 4 and 18 years of age. The group comparison was adjusting therapy based on clinical symptoms with or without spirometry/peak flow without the use of FeNO sputum eosinophils or another biomarker. This model consists of three mutually exclusive non-absorbent states: “no symptoms or asthma controlled”, “suboptimal control without exacerbation”, and “asthma exacerbation” (Fig. 1). In this model, all patients entering in “no symptoms state”, were diagnosed with mild to moderate persistent asthma (all patients receiving inhaled corticosteroids at low doses as maintenance therapy), according to GINA 2021 classification [13]. Then the patients in the simulation move to other states according to transition probabilities entered in the model. The cycle length in this model is 1 week with an analytic horizon of 12 months. All analysis was made from a societal perspective. Given the shorter time, the horizon discount rate was not applied to cost and QALY. Cost-effectiveness was evaluated at a willingness-to-pay (WTP) value estimated in Colombia of US $5180 per QALY gained [14].

### Probabilities of Markov model and utilities

Relative risk, transition probabilities, and QALY were estimated from previously published local studies, Table 1. Data on the relative risk (RR) of FeNO-guided treatment were extracted from metanalysis of randomized controlled trials reported in our previous economic study of FeNO [5]; while RR of tailoring asthma interventions based on sputum eosinophils was extracted from a Cochrane systematic review with meta-analysis [6]. The transition probabilities and QALY were extracted from a previous local study done on asthmatic Colombian children [15]. All estimations were subjected to a probabilistic sensitivity analysis, as detailed below and as recommended by the Consolidated Health Economic Evaluation Reporting Standards (CHEERS) statement because this data does not come from the Colombian population. For all analyses, we used the CI 95% of RR estimated in each paper, while for QALY and transition probabilities; we estiamted the upper and lower range by adding or subtracting 25% to or from the central value defined for the base case.

**Table 1:** Model inputs

Model input	Base case value	Distribution
*Transition probabilities*		
W to S	0.097	β(SD: 0.029)
W to A	0.004	β(SD: 0.002)
S to W	0.817	β(SD: 0.038)
S to A	0.007	β(SD: 0.003)
A to W	0.271	β(SD: 0.044)
A to S	0.052	β(SD: 0.046)
*Utility*		
Well-controlled	0.99	β(SD: 0.016)
Sub-optimal control	0.70	β(SD: 0.072)
Asthma exacerbation	0.31	β(SD: 0.070)
*EO effectiveness*		
Relative risk of reduction of exacerbations	0.57	LogN(SD: 0.20)
*FeNO-SC effectiveness*		
Relative risk of reduction of exacerbations	0.76	LogN(SD: 0.274)

*W* Well-controlled, *S* sub-optimal control, *A* asthma exacerbation

### Cost analysis

The cost of each health state in the model was extracted from a previously published study of costs in 512 children with asthma in Colombia [16], Table 2. In brief, all costs and use of resources in this study were collected from medical invoices and electronic medical records. The direct cost included: medical consultation at the emergency room, specialist referrals, chest physiotherapy, diagnosis support (laboratory, electrocardiogram, x-ray, etc.), medication (oxygen, nebulization, antibiotics, corticosteroids, bronchodilators, etc.), medical devices, accommodation services at intensive care units, and accommodation services in general medical wards. For the valuation of the indirect costs associated with parents’ loss of productivity, the human capital method was used, assuming everyone receives an income of at least legal minimum wage for formal or informal work. The cost-opportunity of the productivity loss at the workplace and the caregiver was assessed based on the minimum wage without including transportation assistance for 2019 (US $229.81 per month) [17].We use US dollars (currency rate: US $1.00 = COP $3000) [18] to express all costs in the study. The incremental cost-effectiveness ratio (ICER) was calculated using the following formulae:

$$ICER = \frac{{\begin{array}{*{20}c} {{\text{Expected annual cost per patient with EO}} - } \\ {{\text{Expected annual cost per patient with FeNO}} } \\ \end{array} }}{{\begin{array}{*{20}c} {QALY\;per\;patient\;with\;{\text{EO}} - } \\ {QALY\;per\;patient\;without\;{\text{FeNO}} } \\ \end{array} }}$$

**Table 2:** Cost used in base case and sensitivity analyses

Model input	Base case value	SA range for one-way sensitivity analyses	Distribution
*Intervention cost*			
FeNO per patient day	2.20	1.20–4.20	γ(SD:1.08)
EO per patient day	9.14	5.15–13.20	γ(SD:4.09)
*Hospitalization cost*			
Daily cost in pediatric ward	95.05	80.23–102.01	γ(SD:8.53)
Hospital length of stay (days)	5.50	4.00–8.00	γ(SD:1.04)
*PICU related cost*			
Daily cost in PICU	406.52	430.26–350.43	γ(SD:18.89)
PICU lenght of stay (days)	10.9	7.75–15.05	γ(SD:3.26)
*Emergency visit prior hospitalization cost*			
Daily cost of emergency ward	64.3	51.19–71.46	γ(SD:19.27)
*Direct medical cost per patient-day*			
Specialist referrals	10.67	10.31–11.01	γ(SD:1.72)
Chest physiotherapy	5.15	4.90–5.39	γ(SD:1.23)
Chest radiography	2.84	2.70–2.98	γ(SD:0.73)
Others diagnostic imaging	0.01	0.0–0.022	γ(SD:0.08)
Complete blood cell counts	1.12	1.05–1.17	γ(SD:0.28)
Other laboratory tests	4.4	4.23–4.47	γ(SD:0.37)
Oxygen	1.37	1.28–1.45	γ(SD:0.41)
Nebulization	16.23	1.28–1.45	γ(SD:4.52)
LEV	1.1	1.07–1.13	γ(SD:0.16)
Antibiotics systemics	1.21	1.11–1.30	γ(SD:0.49)
Systemic o Inhaled Corticosteroids	0.08	0.0–0.90	γ(SD:4.18)
Bronchodilators	0.04	0.03–0.04	γ(SD:0.02)
Other drugs	0.65	0.60–0.68	γ(SD:0.04)
Medical devices	10.24	9.71–10.76	γ(SD:2.66)
*Indirect cost patient-day*	17.24	16.38–18.07	γ(SD:4.30)

Also, we estimated the net monetary benefit (NMB). NMB represents the value of an intervention in monetary terms [19]. NMB is calculated as (incremental benefit × threshold) − incremental cost. Incremental NMB measures the difference in NMB between alternative interventions, a positive incremental NMB indicating that the intervention is cost-effective compared with the alternative at the given willingness-to-pay threshold.

### Sensitivity analyses

To explore the model inputs’ parameter uncertainty, a probabilistic sensitivity analysis was conducted by randomly sampling from each of the parameter distributions (beta distribution in the case of relative risk and utilities, Dirichlet distribution for multinomial data in the case of transition probabilities, and gamma distribution in the case of costs). This process was replicated one thousand times (i.e., second-order Monte Carlo simulation) for each treatment option, resulting in the expected cost-utility. Decision uncertainty is represented in the cost-effectiveness acceptability frontiers. Microsoft Excel® was used in all analyses.

## Results

The main results are presented in Table 2. The base-case analysis showed that compared with FeNO and Standard asthma management, EO was associated with higher costs and higher QALYs. The strategy standard asthma management result dominated (a strategy with higher cost and lower QALYs concerning the others) by FeNO and EO. The expected annual cost per patient with EO was US $1376 (CI 95% US $1376–US $1377) and for FeNO was US $1934 (CI 95% US $1333–US $1334), with a difference of US $42.3 between these strategies. The QALYs per person estimated with EO was 0.95 (CI 95% 0.951–0.952) and for FeNO was 0.94 (CI 95% 0.930–0.940), with a difference of 0.01 between these strategies. The NMB with EO was US $4902 (CI 95% 4900–4904) and for FeNO was US $4841 (CI 95% 4839–4843). The incremental cost-effectiveness ratio of EO was $3566 per QALY gained regarding FeNO. This value is lower than WTP in Colombia of $5180 per QALY gained to declare a health technology as cost-effectiveness (Table 3).

**Table 3:** Case base analysis

Strategy	Cost	Marginal	QUALYs	Marginal	C/E	NMB	ICER
Asthma treatment tailored on sputum esoinophils	$ 1.376	42.3	$ 0.95	0.01		4902	3566
FeNO used in asthma management	$ 1.334		$ 0.94			4841	
FeNO used in asthma management	$ 1.334	− 119.1	$ 0.94	0.02	1419		
Asthma treatment tailored on sputum esoinophils	$ 1.376	− 76.8	$ 0.95	0.03	1446		
Standard asthma management	$ 1.453		$0.92		1573	4755	Dominated

*FeNO* fractional exhaled nitric oxide, *C/E* cost/effectiveness ratio, *Marg C/E* Marginal cost/effectiveness ratio, *QALYs* quality-adjusted life years

### Sensitivity analyses

**Fig. 2:**
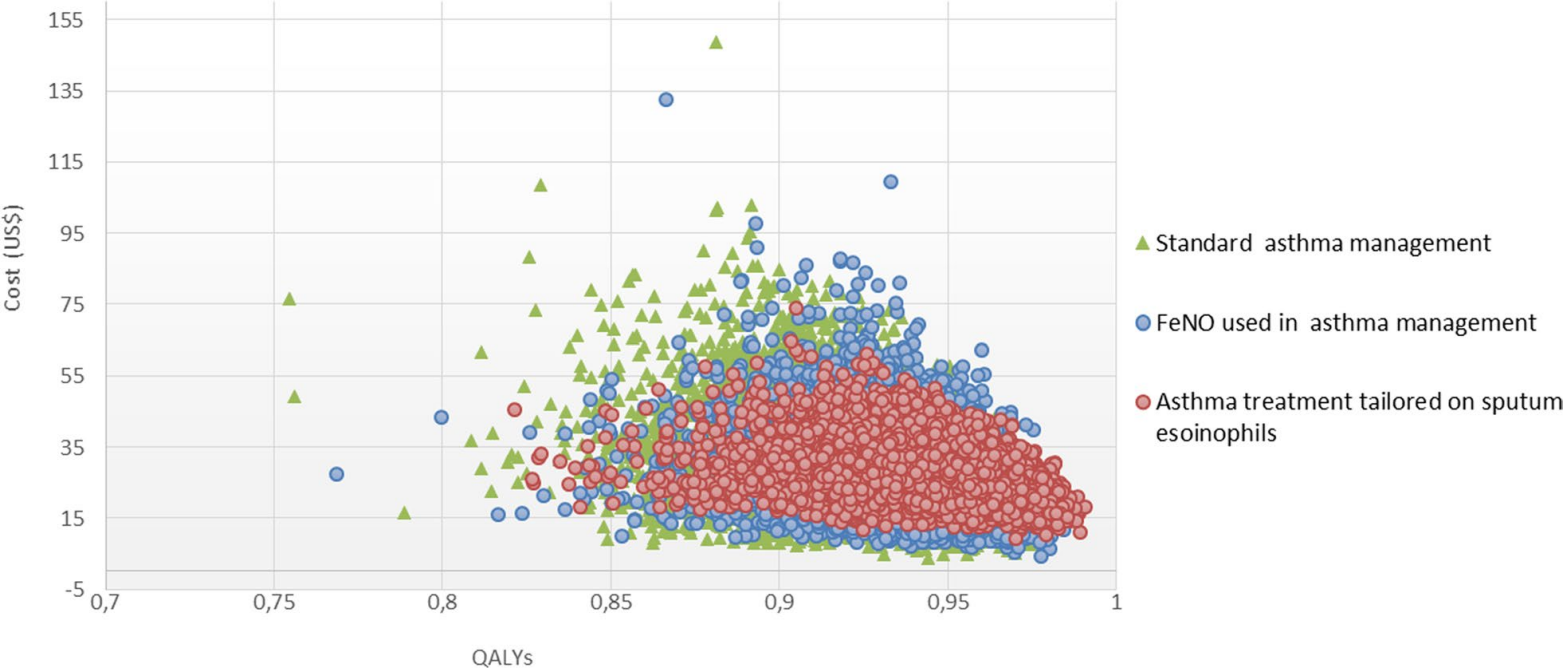
Cost effectiveness plane

**Fig. 3:**
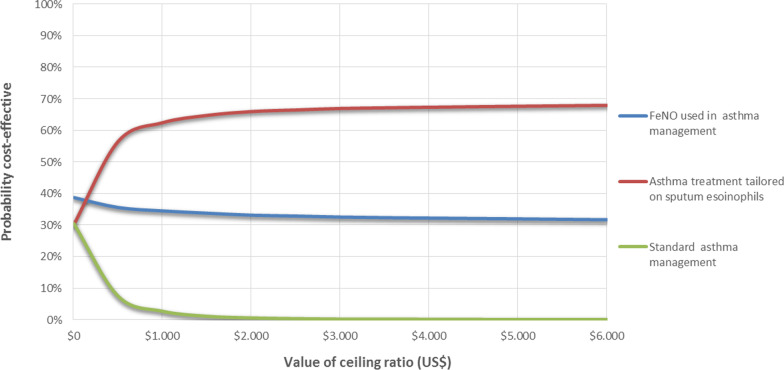
Acceptabilty curve

In the deterministic sensitivity analyses, our base‐case results were robust to variations in utilities, transition probabilities, relative risk, and cost; That is, changing each of the parameters, within the ranges mentioned in the methods section, of cost, utilities, transition probabilities, and relative risk did not change the ICER. The results of the probabilistic sensitivity analysis are graphically represented in the cost-effectiveness plane, Fig. 2. This scatters plot shows that 35% and 33% of simulations of the ICER were in quadrants 2 and 1, while 23% and 8% were in quadrants 4 and 3. The incremental net monetary benefit (INMB) calculated in the second-order Monte Carlo simulation was US $60 (CI 95% US $58 to US $64). This positive value of INMB means that the incremental benefits in monetary terms for the WTP are higher than the incremental costs of this drug in Colombia; thus, this medication can be declared cost-effective. For WTP in Colombia (US $5180 per QALY), EO is cost-effective in 68% of cases versus FeNO as can be seen in the acceptability curve (Fig. 3).

## Discussion

Our study demonstrates that induced sputum-guided management is a strategy cost-effective over FeNO and standard asthma management in Colombia. This conclusion is supported by fact that the incremental cost-effectiveness ratio of EO ($3566) was lower than the willingness-to-pay (WTP) value accepted in Colombia of US $5180 per QALY gained. Always that any health technology has an ICER lower than WTP for the country where this technology is assessed; this technology can be declared as cost-effective [20]. Even more, EO had a positive incremental net monetary benefit concerning FeNO; this means that EO had a more value in monetary terms than FeNO, and always than a health technology had positive incremental NMB indicating that the intervention is cost-effective compared with the alternative at the given willingness-to-pay threshold [19].

Our findings are in-line with previous studies. In our previous publication, we show that EO was associated with a lower total cost (and higher than standard therapy). Induced sputum-guided management has been associated previously with lower cost than standard asthma management ($2265 annual per patient vs. $3369 annual per patient) in adult patients; due to the lower cost of hospital visits (*P* = 0.078), asthma medications (*P* = 0.064) [21]. Indeed, the EO testing has been associated with an increase of cases correctly diagnosed as asthmatic with respect to inhalation challenges with a lower cost per additional correct diagnosis ($3465 vs. $418) [3]. One of the frequent concerns about EO is sampling. Covar et al. Report a sampling success with an adequate sputum sample for analysis in 90 out of 117 children without any adverse event [22]. Similar results have been reported by Lönnkvist and Fleming [7, 8].

To our knowledge, this is the first head-to-head comparison between EO and FeNO, in this case using a mathematical and econometric simulation. Although our study is the first in the pediatric population, it confirms previous findings in the adult population regarding the efficiency of EO for making decisions in asthma management. In our country, these results improve the actual evidence and bring information to the pediatricians and pulmonologists about where strategy is used to guide the management of asthma. Our model was robust in changing the values ​​of the model's utilities, probabilities, and costs using one-way and probabilistic sensibility analysis. EO was always the cost-effective strategy in all value ranges of utilities, probabilities, and costs. These findings in the sensitivity analysis are of cardinal importance in our study because many of the inputs were extracted from literature, which was all hospital-based and undertaken in affluent countries. They also allowed decision-making with an estimated degree of uncertainty in each cost parameter or QALYs per strategy.

Our study has some limitations. The cost data were collected retrospectively. Asthma treatment and the costs in question, including hospital prices, did not markedly change to date. Furthermore, our country has been characterized by having very low price variation in the last 10 years, especially in terms of health services [23]. In addition, we use utilities extracted from the literature and not estimated directly from our population. As was mentioned previously, the reliability and robustness of the results were evaluated by sensitivity analyses. Another limitation is the time horizon in our study of 12 months. We choose this time horizon because clinical trials have not been evaluated effectiveness of FeNO and EO beyond of this time. Therefore, we do not know the cost effectiveness of the tests evaluated beyond the time horizon evaluated. FeNO and EO have been evaluated separately so the inputs are all based on separate evaluations and not using a head-to-head comparison in clinical trial. However to answer this question we use a mathematical model to simulate three cohorts of patients, each receiving FeNO-guided management, EO-guided management, and standard management. At the end, evaluate the costs and benefits measured in quality-adjusted life years gained by each of the alternatives.

## Conclusion

Our study demonstrates that induced sputum-guided management is a strategy cost-effective over FeNO and standard asthma management in Colombia. This evidence that could be used by decision-makers to improve clinical practice guidelines, although it should be replicated in different clinical settings.

## Data Availability

DATABASE BIOMARKERS [Data set]. Zenodo. https://doi.org/10.5281/zenodo.5992128.

## Abbreviations

FeNO: Fractional exhaled nitric oxide
FeNO-SC: SC with FeNO monitoring to guide asthma management in children
QALYs: Quality-adjusted life years
RCT: Randomized clinical trials
